# Conserved features of recombination control in vertebrates

**DOI:** 10.1371/journal.pbio.3002959

**Published:** 2025-01-07

**Authors:** Linda Odenthal-Hesse

**Affiliations:** Research Group Meiotic Recombination and Genome Instability, Max Planck Institute for Evolutionary Biology, Plön, Germany

## Abstract

This Primer explores a recent study in PLOS Biology on the epigenetic recombination regulator PRDM9 in salmonid fish, which reveals that its function has been preserved across vertebrates for hundreds of millions of years, with rapidly evolving DNA-binding domains being a defining attribute.

## Teaser

A recent study in *PLOS Biology* on the epigenetic recombination regulator PRDM9 in salmonid fish reveals that its function has been preserved across vertebrates for hundreds of millions of years, with rapidly evolving DNA-binding domains being a defining attribute.

The discovery of the role of PRDM9 in orchestrating meiotic recombination was a breakthrough in understanding recombination regulation. The methyltransferase domain of PRDM9 places signaling beacons on histone residues, trimethylating histone 3 lysine 4 (H3K4me3) and histone 3 lysine 36 (H3K36me3), thus creating a double-positive mark outside the functional elements destined for transcription [1]. PRDM9 targets hotspots via its DNA-binding domain, which binds to specific DNA motifs found across the genome. As such, PRDM9’s role is to direct recombination away from promoters of meiotic transcription.

Meiotic recombination is initiated through double-stranded DNA breaks at the PRDM9 motif, which are repaired by an exchange of genetic material from the homologous chromosome. The sites of genetic exchange are concentrated in so-called recombination hotspots. If DNA-binding motifs differ due to sequence divergence between homologs, the motif with stronger binding affinity will preferentially receive an initiating double-stranded DNA break. As breaks are repaired by copying information from the unbroken homolog, over time, the initially stronger binding motifs (which will be cut more often) are preferentially replaced by weaker motifs (which are cut less often) [2]. As the strengths of hotspot motifs across the genome decrease over time, this phenomenon has been called hotspot erosion.

Paradoxically, new hotspots are activated at novel sites genome-wide due to the rapid evolution of the DNA-binding domain of the PRDM9 protein. This rapid evolution is due to 2 factors: the DNA-binding domain of PRDM9 is encoded by a hypervariable minisatellite with high de novo repeat instability [3], and there is strong positive selection on the positions involved in DNA binding.

The PRDM9 protein was initially thought to be essential for fertility, but, although a complete gene was present in the common ancestor of all vertebrates, PRDM9 has been lost at least 13 times independently, including in canids, amphibians, crocodilians, birds, and several fish species [4]. At least in yeast, plants, birds, and dogs, PRDM9-independent recombination shows unifying characteristics of high recombination rates at sites of open chromatin at promoter regions, including transcription start sites and at promoter-associated CpG islands.

PRDM9-dependent and independent recombination pathways can apparently coexist in scaly reptiles, as corn snakes use both promoter-associated hotspots and PRDM9-mediated recombination hotspots [5]. Furthermore, rattlesnakes preferentially place meiotic DSBs at promoter regions despite the presence of functional PRDM9 [6]. Therefore, a major gap in our understanding has been whether non-mammalian taxa with PRDM9 use it to drive the location of recombination events or whether hotspot determination by PRDM9 might be somewhat relaxed outside of mammals.

To address this knowledge gap, Raynaud and colleagues investigated the role of the *Prdm9* gene in determining the location and rapid evolution of meiotic recombination hotspots in salmonid fish [7]. Salmonids are a diverse family of teleost fish that likely have PRDM9-mediated recombination, as the authors could show several salmonid species that possess a full-length copy of PRMD9 and all genes that tend to co-evolve with PRDM9. Multiple copies of PRDM9 are present in salmonids, originating from several whole-genome duplication events, and distinct lineages retained different paralogues as functional copies.

**Fig 1:**
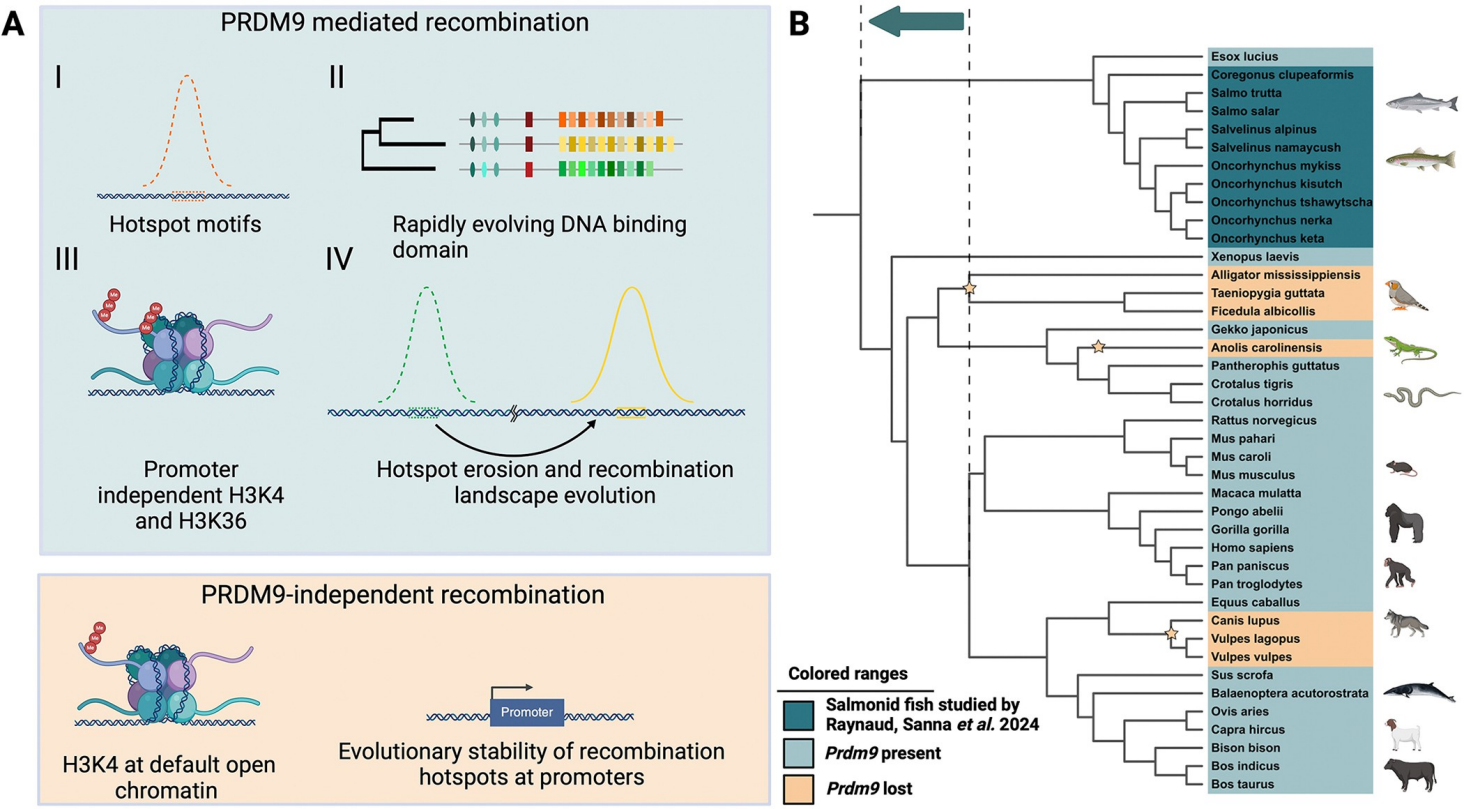
Functional PRDM9 in salmonids has 4 hallmark features. (**A**) (**I**) Recombination hotspots that share common motifs, (**II**) rapid evolution and high variability in its DNA binding domain, (**III**) a double positive epigenetic H3K4 and H3K36 trimethylation signal, (**IV**) positional variation in hotspots between populations. PRDM9-independent recombination occurs at promoters with characteristic H3K4 trimethylation, and the positions of hotspots show evolutionary stability. (**B**) As the distinctive characteristics of PRDM9 were previously only known to exist in mammals, the results of Raynaud and colleagues thereby move the evolutionary origin of the dynamic hotspot placement by the PRDM9 protein to an earlier time than previously believed, indicated by an arrow connecting the dashed lines in a phylogenetic tree of representative species, where PRDM9 losses identified by [4] are indicated by stars. Created in BioRender. ODENTHAL-HESSE, L. (2024) https://BioRender.com/l11h793.

Rainbow trout (*Oncorhynchus mykiss*) and Atlantic salmon (*Salmo salar*) showed high diversity in the DNA-binding zinc finger domain of the full-length PRDM9 paralog. High within-population diversity has been shown for mammals including humans, whales, and mice. In rainbow trout, recombination also clustered away from promoter regions, and hotspot sites were double-positive for histone modifications H3K4me3 and H3K36me3. The location of hotspots was dependent on the PRDM9 genotype of the fish, evidence for a direct role of PRDM9 in the specification of recombination hotspots in salmonids. Population-scaled recombination hotspot positions did not overlap fully, providing further evidence of recombination hotspot turnover and supporting the hypothesis of positive selection driven by the evolutionary “arms race” between PRDM9 and its binding sites. Taken together, the data collected by Raynaud and colleagues highlights that the 4 hallmark characteristics of PRDM9-mediated recombination (Fig 1) are present in salmonid fish and, therefore, not exclusive to mammals.

We still need to understand what makes an organism use the PRDM9-mediated pathway, which organisms can use promoter-associated open chromatin instead, and under which conditions. In particular, it remains to be studied whether these processes are driven by binding site erosion, for example, if the default pathways are activated when a critical threshold of low hotspot counts is crossed, and whether hotspot erosion is an evolutionary driver for activating efficient repair strategies in each lineage. This raises the question of how efficient DNA repair at functional elements and promoters can be achieved in different lineages and whether all these organisms can reactivate the same repair pathway—or whether each lineage has evolved lineage-specific strategies.

It will likely take studies in diverse organisms across the Tree of Life to understand how and why PRDM9-directed recombination, a genetically unstable mechanism, evolved in the first place.
